# Autoimmune Responses in Oncology: Causes and Significance

**DOI:** 10.3390/ijms22158030

**Published:** 2021-07-27

**Authors:** Halin Bareke, Pablo Juanes-Velasco, Alicia Landeira-Viñuela, Angela-Patricia Hernandez, Juan Jesús Cruz, Lorena Bellido, Emilio Fonseca, Alfonssina Niebla-Cárdenas, Enrique Montalvillo, Rafael Góngora, Manuel Fuentes

**Affiliations:** 1 Department of Pharmaceutical Biotechnology, Faculty of Pharmacy, Institute of Health Sciences, Marmara University, Istanbul 34722, Turkey; halin.bareke@gmail.com; 2 Department of Medicine and General Cytometry Service-Nucleus, CIBERONC CB16/12/00400, Cancer Research Centre (IBMCC/CSIC/USAL/IBSAL), 37007 Salamanca, Spain; pablojuanesvelasco@usal.es (P.J.-V.); alavi29@usal.es (A.L.-V.); angytahg@usal.es (A.-P.H.); emontalvillo@usal.es (E.M.); rgongora@usal.es (R.G.); 3 Medical Oncology Service, Hospital Universitario de Salamanca-IBSAL, 37007 Salamanca, Spain; jjcruz@usal.es (J.J.C.); lbellido@saludcastillayleon.es (L.B.); efonseca@usal.es (E.F.); 4 Department of Nursing and Physiotherapy, Faculty of Nursing and Physiotherapy, University of Salamanca, 37007 Salamanca, Spain; id00712106@usal.es; 5 Proteomics Unit, Cancer Research Center (IBMCC/CSIC/USAL/IBSAL), 37007 Salamanca, Spain

**Keywords:** autoantibodies, solid cancers, cancer immunotherapy, biomarker, autoimmunity, immune-related adverse effects, tumor antigens

## Abstract

Specific anti-tumor immune responses have proven to be pivotal in shaping tumorigenesis and tumor progression in solid cancers. These responses can also be of an autoimmune nature, and autoantibodies can sometimes be present even before the onset of clinically overt disease. Autoantibodies can be generated due to mutated gene products, aberrant expression and post-transcriptional modification of proteins, a pro-immunogenic milieu, anti-cancer treatments, cross-reactivity of tumor-specific lymphocytes, epitope spreading, and microbiota-related and genetic factors. Understanding these responses has implications for both basic and clinical immunology. Autoantibodies in solid cancers can be used for early detection of cancer as well as for biomarkers of prognosis and treatment response. High-throughput techniques such as protein microarrays make parallel detection of multiple autoantibodies for increased specificity and sensitivity feasible, affordable, and quick. Cancer immunotherapy has revolutionized cancer treatments and has made a considerable impact on reducing cancer-associated morbidity and mortality. However, immunotherapeutic interventions such as immune checkpoint inhibition can induce immune-related toxicities, which can even be life-threatening. Uncovering the reasons for treatment-induced autoimmunity can lead to fine-tuning of cancer immunotherapy approaches to evade toxic events while inducing an effective anti-tumor immune response.

## 1. Introduction

The self- vs. non-self-discrimination was long considered to be the sole determinant for immune activation and tolerance. As a safeguard mechanism against autoimmunity, the immune system was thought to be prevented from turning its destructive power toward itself by only recognizing non-self antigens. Since cancer cells are formed from the body’s own cells, the immune system has been deemed to be powerless against these treacherous, but nevertheless self-cells. However, this paradigm fails to account for many aspects of immune responses including the apparent lack of immune reactions to the commensal microorganisms found ubiquitously in the body. The self vs. non-self dichotomy has, then, been revisited with the harmful vs. harmless distinction. The immune system is trained to distinguish between harmful and harmless molecules (albeit, they are mostly self-antigens) through central and peripheral tolerance, the microbes encountered very early in life, and whether the milieu is pro-immunogenic or anti-immunogenic [1]. This shift in paradigm has created the possibility for the existence of anti-tumor immune responses against harmful self-cells. The elevated risk of cancer in immunosuppressed individuals and in immunocompromised severe combined immunodeficiency (SCID) mice corroborates the importance of the immune response in cancer [2]. Under the right pro-immunogenic conditions, the immune cells can, indeed, respond to tumor cells. However, autoimmune responses can concomitantly arise during those anti-tumor responses.

Reinvigorating the immune system against tumor cells in the form of oncological immunotherapy has dominated the cancer therapy field in the last decade [3]. The immune system’s hand is weakened in the fight against a tumor by poor antigenicity of tumor-associated antigens (TAAs), inefficient antigen presentation in the deficit of co-stimulatory molecules, T cell exhaustion, and the immunosuppressive tumor microenvironment (TME) [4]. Among immunotherapeutic approaches, releasing the ‘brakes’ on immune cells to enable their attack on a tumor mass using immune checkpoint inhibitors (ICI) has shown remarkable clinical results in different types of solid cancers, such as renal cell carcinoma and advanced melanoma [5]. However, ICI, as well as some other immunotherapy approaches such as cytokine administration, can stimulate the immune system non-specifically, and hence, they can also trigger the activation of self-reactive lymphocytes. Therefore, immune-related adverse effects (irAEs) such as skin lesions, colitis, and thyroiditis might occur due to an autoimmune attack, limiting the clinical benefit of these treatments [6]. As these therapies are finding their place in the clinic rather quickly, evading irAEs and stratifying patients at a risk of developing immune toxicities are becoming particularly important. To this end, it is important to delineate the mechanistic underpinnings of these autoimmune attacks.

As the path from cancer to the autoimmune response is not only caused by therapeutical treatment, other paths, especially due to the ‘self’ nature of the cancer cells, co-exist with the same autoimmune outcome. In fact, there is a high concentration of self-antigens released by conventional therapies in most solid tumors as well as some TAAs that closely resemble self-antigens, leading to autoantigen cross reactivity. Cancer and the autoimmune response can both be conceptualized under the umbrella of ‘immune dysregulation’. In the case of cancers, the immune system fails to clear the altered dangerous ‘self’ cells in a case of ‘abortive autoimmune response’ [7]. During the autoimmune response, the immune system launches a response on innocuous components of self-cells. Even though these two immune imbalances appear to be the opposite of each other, they can still continuously feed and bear each other. The constant attack on the self-cells and the chronic inflammation seen in autoimmunity can promote tumorigenesis through multiple mechanisms including increased cell division and DNA-damaging oxidative stress [8]. Given also the plasticity of lymphocytes, as exemplified by the conversion of inflammatory Th17 cells into suppressor regulatory T cells (Tregs), the two extremes are closer than anticipated in a normal immune response. Furthermore, from a functional point of view, autoantibodies (aAbs) from the autoimmune response may sometimes actively promote tumorigenesis and tumor progression [9]. However, in some tumors such as melanoma, the presence of aAbs can signal an effective anti-tumor response and be associated with better disease outcomes [10]. Moreover, it is well-described that autoantibodies are present even before the onset of overt cancer. In some cases, there might be autoimmune manifestations in the form of paraneoplastic syndromes in the early stages of cancer [11]. If these autoimmune responses are detected quickly, they can help with life-saving early diagnosis of cancer. Recent studies, including one from our research team, have also raised the possibility that the autoantibody profiles in cancer can be used as biomarkers for diagnosis and discrimination between metastatic and non-metastatic disease [12].

Overall, understanding the profile and the mechanism of the autoimmune response observed in solid tumors would shed light on basic mechanisms of immune tolerance, would help to refine cancer immunotherapy approaches to minimize immune-related toxicities, and might yield much-needed cancer biomarkers for early cancer diagnosis and accurate prognosis. Herein, we will focus solely on the observed autoimmune responses in solid malignant tumors. Hematological malignancies warrant their own dedicated discussion regarding the links between autoimmunity and these malignancies because both the malignant cells and the cells involved in autoimmune responses are the same cell population, namely lymphocytes. In the following sections, the anti-tumoral immune responses and autoimmune responses, mainly in the form of aAbs and irAEs, that are detected in solid cancer patients, as well as their possible causes and significance, will be discussed.

## 2. Immune Tolerance and Anti-Tumoral Immunity

Due to the self-nature of cancer cells, central and peripheral immune tolerance mechanisms reduce both the chance of occurrence and the effectiveness of anti-tumor immunity. These mechanisms are in place to prevent the maturation and activation of self-reactive lymphocytes. Central tolerance takes place in the primary lymphoid organs, namely the bone marrow and the thymus. In the thymus, thymocytes present a variety of self-antigens from the tissues all over the body to the developing T cells thanks to the thymic-specific expression of certain genes such as *AIRE* [13]. Those T cells that recognize self-peptides are negatively selected in the thymus by undergoing apoptosis during thymic education. Similarly, in the bone marrow, developing B cells that bind to self-antigens either edit their B cell receptors (BCR) to change receptor specificity or die by apoptosis [14].

The self-reactive lymphocytes that have survived central tolerance and reached maturation are ‘subdued’ in periphery by regulatory cells such as CD4+CD25+FOXP3+ Tregs, by anergy induction (functional unresponsiveness), by lack of co-stimulation, and by induction of cell death (i.e., deletion). However, even in a normal physiological state, central and peripheral tolerance is far from perfect, and self-reactive T and B cells are found in healthy individuals [15]. Therefore, rather than eliminating all of the self-reactive lymphocytes, these mechanisms might only reduce their number and raise their activation threshold. Under certain conditions such as a high concentration of self-antigen and a highly proinflammatory cytokine milieu, these autoreactive lymphocytes might assemble a specific immune response, including in cancer settings.

Evading immune response is required for the cancer cells’ survival, and thus, immune evasion has been recognized as one of the hallmarks of cancer [16]. Tumor mass has to establish an ‘immune-privileged’ site for itself by adopting tactics that are similar to natural immune privilege mechanisms including the expression of immunosuppressive cytokines, downregulation of major histocompatibility complex (MHC) molecules, induction of lymphocyte apoptosis by molecules such as immune checkpoint molecules, PD-L1, prevention of T cell stimulation by another immune checkpoint protein, CTLA-4, recruitment of Tregs and other immunosuppressive cell populations such as myeloid-derived suppressor cells (MDSC), and inhibition of effector leukocyte infiltration [17]. The major suppressive lymphocytes, CD4+CD25+FOXP3+ Tregs, can suppress effector T cell activity by inducing cytolysis by granzyme B/perforin and apoptosis through death receptors, sweeping the IL-2 from the environment, secreting immunoregulatory cytokines such as IL-10 and transforming growth factor-β (TGF-β), inhibiting the stimulatory function of DCs, and by the generation of extracellular adenosine [18]. The importance of Tregs in cancer has been revealed through the prognostic significance of Treg presence in solid cancers. In a meta-analysis that included 15,512 cancer cases with 17 different types of solid cancer, high FOXP3+ Treg-density in tumor samples was associated with a lower overall survival rate in the pooled data [19]. Another immunoregulator cell population, MDSCs, are myeloid-derived immature cells that can suppress T cells via several mechanisms including cell contact, tryptophan deprivation by indoleamine 2,3-dioxygenase (IDO) expression, L-arginine depletion by arginase, Treg recruitment, and reactive oxygen species (ROS) generation [20]. A meta-analysis that included 1864 cancer patients showed that MDSC was a bad prognostic factor that shortened overall survival [21]. These data support the importance of anti-tumor responses in shaping the disease course in cancer, and demonstrate how the cell populations that act as safeguards against autoimmunity can hamper effective anti-tumoral immune responses.

The immune system can recognize cancer cells through TAAs or by self-reactive lymphocytes. Therefore, the lines between autoimmune response and cancer immunity are blurred such that the immune tolerance might be compromised during anti-tumoral immune responses. TAAs can be neoantigens that include aberrantly expressed or modified self-antigens, or oncofetal antigens. The players that counteract the immunosuppressive TME are mainly the components of cellular immunity. They include tumor-lysing populations such as natural killer (NK) cells and CD8+ cytotoxic T lymphocytes (CTLs). Lysing of the tumor cells by these lymphocytes releases a high concentration of self-antigens and proinflammatory molecules that can potentially trigger autoimmune responses. IFN-γ secreted by NK cells also promotes dendritic cell maturation and secretion of IL-12. Mature dendritic cells are pivotal in presenting both endogenous and exogenous (by cross presentation) TAAs to the CD8+ T cells and providing the right costimulatory signals (i.e., signal 2) for T cell activation [22]. In the CD4+ T cell compartment, Th1 polarization that activates M1 macrophages is important in killing cancer cells. M1 macrophages, in turn, promote the recruitment of CTLs and Th1 cells. Accordingly, the presence of tumor-infiltrating lymphocytes (TIL) is usually correlated with a better prognosis. As an illustrative example, a meta-analysis of 43 studies on the association between TIL and colorectal cancer prognosis showed that high TIL numbers and CD3+ T cell density were associated with disease-free survival as well as overall survival [23].

Compared to T cells, much less is known about tumor-infiltrating B cells (TIBs). Their importance in the immune control of cancer remains to be fully uncovered. TIBs might promote T effector function by cytokines or by antigen presentation. In addition, antibodies produced by TIBs can bind to their cognate antigens on tumor cells, modifying the function of the antigens. They can also act as an opsonin, activate the classical pathway of complement, or induce antibody-dependent cellular cytotoxicity (ADCC) [24]. TIBs could also have a suppressive effect through subduing T cell responses by recruiting Tregs or by secreting immunomodulatory cytokines such as IL10 or TGF-β [25]. A meta-analysis that included 19 solid cancers found that CD20+ TIBs were mostly associated with a good prognosis; however, specific markers for different subtypes of B cells are required to shed more light on this data [24]. In the same study, the prognostic value of the plasma cells was found to be less straightforward. When IgG4 was used as a plasma cell marker, it was associated with negative outcomes in two out of three cancer types (gastric and pancreatic ductal adenocarcinoma) studied [24]. When the immunoglobulin kappa-light chain was used as a plasma cell marker, the presence of plasma cells was mostly associated with better outcomes, albeit in different cancers than cited above.

B cell-associated autoimmune responses are noted frequently in the serum of patients with solid cancers. Furthermore, a study has shown that 84% of breast cancer tissue contains autoantibodies secreted by TIBs [26]. Therefore, autoantibodies are found both in peripheral blood and in situ. Knowing more about cancer-associated B cell responses and the antigen specificity of antibodies, including aAbs, is important because antibodies are stable and easy to assay, as opposed to T cell functional assays, and can help in early diagnosis or the stratification of patients for treatment choice or prognosis as well as helping in the development of new cancer immunotherapy approaches.

## 3. Origins of Autoimmune Responses in Oncology

**Figure 1:**
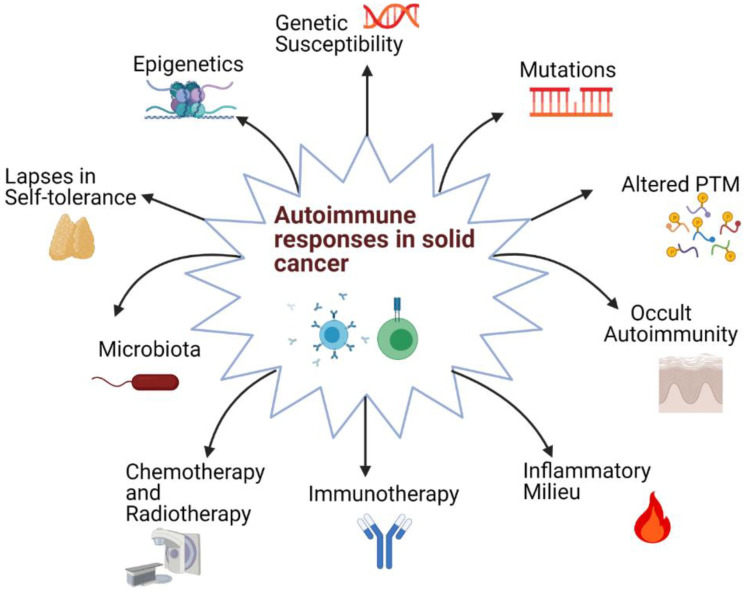
The probable causes of autoimmune responses observed in solid cancers. Various factors ranging from mutations to therapy-induced autoimmunity can lead to autoimmune responses. The multifactorial nature of this phenomenon can contribute to the extent of variation observed in autoimmune responses in patients (PTM: Post-translational modification).

The precipitating factors for autoimmune responses in cancer can be multifactorial and include a combination of host genetic factors, the inflammatory milieu in the host, the nature of TAAs, and the effects of cancer therapy interventions (Figure 1).

### 3.1. Shared Genetic Factors

Solid tumors and autoimmune diseases both involve a genetic component; thus, a question arises as to whether there is a shared genetic component between tumorigenesis and autoimmune susceptibility. The shared genetic component between autoimmunity and cancer could possibly be in the form of loss-of-function and gain-of-function gene mutations as well as single nucleotide polymorphisms (SNPs). These changes can be in protein-encoding genes that are important both for cancer and autoimmunity progression such as apoptosis.

The evasion of apoptosis is one of the hallmarks of cancer, and many factors, including mutations in anti-apoptotic and proapoptotic genes, enable this evasion to take place. The role of apoptosis in autoimmunity is more complex, and it can be involved in the evasion of central and peripheral tolerance mechanisms by self-reactive lymphocytes. Hence, such genetic factors can promote the survival of autoreactive lymphocytes by sparing them from deletion during negative selection and from activation-induced cell death. Therefore, genetic alterations that lead to reduced apoptosis can link cancer and autoimmune responses. *TP53* is a prototypical proapoptotic gene that is mutated in many different solid tumors [27]. The protein encoded by this gene, p53, is pivotal in DNA repair, cell cycle arrest, and the induction of apoptosis in the case of excess DNA damage to conserve genomic stability. Hence, mutations that inactivate p53 promote genomic instability, which is also a hallmark of cancer and enables the tumor to adapt quickly to survive multiple assaults, both from treatments and the immune response. aAb responses against this protein have also been detected in some forms of cancer including pancreatic cancer [28]. Interestingly, mutations in *TP53* have been shown to increase autoimmune susceptibility in multiple strains of mice [29,30]. When T cells are deficient in p53, the reduction of FOXP3+ Tregs has been observed, suggesting a possible link between p53 and Treg induction [18,28]. In human studies, it has been shown that rheumatoid arthritis (RA) patients have lower p53 expression and elevated Th17 numbers, suggesting that in Treg/Th17 plasticity, p53 can shift the balance toward Tregs [31]. SNPs in *TP53* are also associated with a higher risk for some autoimmune diseases, such as inflammatory bowel disease [32]. A recent study that investigated the effect of p53 peptides on the peripheral blood mononuclear cells of type I diabetes patients showed that even though p53 peptides increased CD8+ Treg numbers, they also increased T effector cells [33]. These data raise the possibility that p53 and autoimmune responses might not be linked via an immune mechanism or that p53 is involved in the initial stages of pathophysiology [33]. Interestingly, aAbs against the negative regulator of p53, MDM2, have been detected in the serums of patients with lung cancer and autoimmune diseases, namely systemic lupus erythematosus (SLE) and Sjogren’s syndrome [34,35,36]. Furthermore, these aAbs have been suggested as biomarkers for both cancer and autoimmune diseases [37,38]. The similar aAb profile between autoimmune diseases and cancer for the p53 pathway and the reduced function of p53 observed in both types of diseases can thus point to a shared, gene-based factor.

Akt (protein kinase B, PKB) is a serine/threonine protein kinase and a key mediator in the phosphoinositide 3-kinase (PI3K) pathway. Akt promotes proliferation and migration while suppressing apoptosis. Not surprisingly, its levels are increased in many different types of solid cancers including breast and pancreatic cancers [39]. The importance of the PI3K pathway is underscored by the fact that the gene coding for a subunit of PI3K, *PIK3CA*, was found to be the second-most-commonly mutated gene after *TP53* in a study of 12 different cancer types in the Cancer Genome Atlas [27]. *Akt* is a protooncogene, and when the genomic data from patient samples of 32 different cancer cell types were analyzed, *AKT1* was found to be mutated in 1% and amplified in 3% of 11,219 analyzed cases [40]. The pro-survival effects of Akt might also help lymphocytes evade central and peripheral tolerance. In a study where transgenic mice had T cells expressing Akt under the control of human CD2 promoter, both B and T cells accumulated in the lymph nodes and spleen, and T cells had a higher activation state with resistance to Fas-mediated apoptosis, and the mice exhibited systemic autoimmunity [41]. PI3K signaling has also been shown to inhibit in vitro Foxp3 expression and Treg differentiation in mice [42]. Higher activity by Akt in mouse Tregs due to the selective knock out of the negative regulator, *PTEN*, led to lymphoproliferative disease, renal failure, and the inability to resolve autoimmune encephalomyelitis, all of which indicated lower Treg activity [43]. A study on humans also showed that, pemphigus vulgaris patients had higher mRNA levels for the components of PI3K pathway, including Akt and its phosphorylated form, and a higher Th2/CD4+ T cell ratio than the controls [44]. Overall, the activation of this pathway confers a pro-survival advantage to cancer cells and effector T cells, whereas it decreases Treg differentiation, showing yet another pathway that is involved in both autoimmunity and solid cancers.

Another molecule that promotes cell survival is Bcl-2. Bcl-2 is a part of the intrinsic apoptotic pathway and is located on the mitochondrial membrane [45]. Bcl-2 inhibits apoptosis induced by the other BCL-2 family members, Bax and Bak, and hence, it is important for cell survival. *Bcl-2* is mutated in some solid cancers such as skin cancer and lung adenocarcinoma [46]. It has been shown that Bcl-2 overexpression protects various cancer cells from apoptosis [47]. Certain Bcl-2 genotypes have also been shown to be associated with lupus [48]. The role of anti-apoptotic Bcl-2 in peripheral tolerance was shown when Bcl-2 overexpression prevented the apoptosis of ovalbumin (OVA)-reactive CD8+ T cells in transgenic mice where OVA was a self-antigen [49]. Consequently, genetic events that lead to *Bcl-2* overexpression can promote both cancer and autoimmune responses.

The expression of genes can also be altered by epigenetic changes. Epigenetics refers to the reversible, and sometimes heritable, changes to DNA and/or chromatin that can affect gene expression without altering the nucleotide sequence. These epigenetic modifications can be in many forms including DNA methylation patterns and histone modifications [50]. Epigenetic processes are pivotal for many other processes including cell differentiation, proliferation, and survival. Enzymes such as DNA methyltransferases (DNMT), histone methyltransferases (HMT), and histone deacetylases (HDACs) create modification patterns that alter levels of gene expression, as exemplified by the trimethylation of Lysine 4 on histone 3 (H3K4 3me) that leads to transcription activation [51]. The epigenome changes drastically in cancer settings, affecting the DNA, RNA and histone components [52]. These changes affect the transcriptional status of genes and overall chromosomal stability [52]. Chronic inflammation is considered one of the crossroads between autoimmunity and cancer. In a meta-analysis of epigenome-wide association studies (EWAS) on the methylation of DNA and C-reactive protein (CRP) levels (as an indicator of low-grade inflammation), it was shown that the methylation patterns of many CpG island sites were associated with CRP levels for people of both European and African-American ethnicity [53]. Chronic inflammation can also induce epigenetic changes such as the aberrant hypermethylation of CpG islands, which lead to the transcriptional inactivation of tumor suppressor genes [54]. As the scope of this review is primarily the road from solid cancers to the autoimmune response, it is of note that aAbs against nucleosomes are found in autoimmune diseases. In particular, 88% of SLE patients had anti-nucleosome antibodies [55]. It has been put forth that the apoptotic epigenetic signature of nucleosomes increases their immunogenicity [52]. During cancer treatment, the induction of massive apoptosis by various treatment regimens might, thus, prompt the generation of anti-nucleosome aAbs.

MHC molecules are crucial for antigen presentation and hence, for T cell activation. MHC gene loci are highly polymorphic, with HLA-B being the most polymorphic locus in the human genome. MHC molecules dictate the epitopes that are presented to T cells. It has been suggested that some MHC molecules can present epitopes with close resemblance to self-peptides, which may lead to the activation of autoreactive T cells. Variants in the MHC loci are also strongly associated with many different autoimmune diseases, such as the strong *HLA-B27* association of ankylosing spondylitis [56]. Given the highly polymorphic nature of the MHC loci and their importance in antigen presentation, such an association is not surprising, even though the direct mechanistic link has not yet been clearly elucidated. MHC alleles have also emerged as important in the autoimmune responses precipitated by ICI. Interestingly, the type I diabetes risk-associated MHC class 2 allele, HLA-DR4, is more prevalent in patients who develop diabetes as an irAE in anti- PD-1 and PD-L1 therapy than what is normally found in the US Caucasian population [57]. More studies are required to identify the reasons for this association, yet in a multiple hit theory for autoimmunity, the ICI might be another hit that precipitates autoimmune responses in genetically susceptible individuals.

### 3.2. Microbiota

Recent advances in -omics, including metagenomics, have revealed the pivotal role of microbiota in health and disease. Consequently, the human body cannot be regarded separately from its microbiota. Microbiota contain commensal bacteria, viruses, protozoans, fungi, and archaea that mainly colonize the mucosal surfaces and the skin. Interactions among the members of the microbiota and with the host have the potential to shape the course of infections and the immune tolerance landscape of the host.

Microbiota, especially gut microbiota, act as physical and biochemical barriers for the immune system and as shapers of the inflammatory response through training the immune system. The immunological effects of microbiota are thought to be mediated by microbial metabolites as well as immune components such as cytokines and ‘gut-trained’ immune cells [58]. As an example, short-chain fatty acids (SCFA) such as butyrate produced by intestinal microbiota are important in tolerance induction by promoting Treg differentiation [59]. Gut dysbiosis, defined as an imbalance in the gut microbial community, has been linked to many diseases including Crohn’s disease, RA, and respiratory diseases [60,61].

Microbiota are also important in shaping immune-based responses in cancer. Antibiotics can cause dysbiosis and disrupt the commensal microbiota. The importance of microbiota in the ICI treatment response was highlighted when administering antibiotics before ICI treatment reduced the survival benefit from ICI in renal cell carcinoma [62]. The over-representation of *Akkermansia muciniphila* species in the gut microbiota has been shown to be significantly associated with favorable outcomes upon ICI in renal cell carcinoma and non-small cell lung carcinoma patients [4]. This favorable outcome was associated with higher interferon gamma (IFN-ɣ) release by Th1 in the presence of *A. muciniphila* [4]. Primary resistance to anti PD-1 therapy is an important problem, and biomarkers associated with treatment response are under intense investigation. The microbiota content has emerged as an important predictor of treatment response [4]. Recently, a clinical trial was conducted wherein fecal microbiota transplant (FMT) was performed on metastatic melanoma patients who were refractory to anti PD-1 therapy [63]. FMT involves the introduction of the normal flora found in the stool of a donor into the colon of a recipient to transform their gut microbiota. In this study, patients who responded to anti-PD-1 therapy and were disease-free were used as donors, and it was shown that ICI resistance was reversed in 6 out of 15 patients due to the change in the gut [63].

The phyla of bacteria found in the gut microbiota also seem to protect from or promote the induction of irAEs as a result of ICI therapy. It was shown that having more bacteria from the Bacteroidetes phylum protected from colitis in CTLA-4 therapy, whereas *Faecalibacterium* raised the risk of ipilimumab treatment-associated colitis [64].

The role of the microbiota in the link between anti-tumor immunity and autoimmune responses could be multifaceted. The microbial communities have been deemed ‘extended self’ cells, and they can shape the antigens to which the immune system is tolerant [65]. Toll-like receptor 2 (TLR-2), which is one of the main pattern recognition receptors, upon engagement with polysaccharide A antigen from the Bacteroidetes phylum, promotes an anti-inflammatory environment by inducing Tregs and secretion of IL-10. In this way, commensal bacteria can establish a symbiotic existence with the human host. The relative abundance of some species as well as changes in abundance can change the tolerance landscape. Changes in the microbial communities can lead to immune responses with potential cross-reactivity with self-antigens, as exemplified by Th17 activation against some specific bacterial antigens with cross-reactivity to the myelin leading to demyelination [65]. A similar molecular mimicry between microbial peptides and self-antigens has been shown for type II collagen and peptides from certain members of the microbiota [66]. Microbial enzymes such as transglutaminases can also aberrantly modify human proteins, rendering them immunogenic, which can induce both anti-tumor immune and autoimmune responses [67].

Furthermore, the microbial communities can shape the polarization of immune cells, especially CD4+ T cells, as well as the type of immune cells recruited for the immune response. IL-17-secreting Th17 cells have emerged as important in mucosal immunity and in keeping the members of the microbiota under control to prevent overgrowth. Tregs and Th17 cells require a common cytokine, TGF-β, for their polarization from CD4+ T cells. These two helper T cell subtypes can transdifferentiate based on the cytokine milieu. As an example, FOXP3+ Tregs can acquire a Th17 phenotype in the presence of IL-6 and IL-23 [65]. Therefore, immune activation through Th17 and immune suppression are not far from each other, and it has also been demonstrated that tumor-infiltrating Th17 cells can be converted to Tregs in immune-suppressive TME [65]. In mouse models, microbiota species that skew T helper differentiation toward Th17 have shown to worsen autoimmunity, and in turn, IL17A-deficient mice are protected from experimental autoimmune encephalomyelitis due to changes in gut microflora [65,68] In terms of cancer, IL-17 has been shown to have both pro- and anti- tumorigenic effects based on the cancer type. On one hand, IL-17 can induce angiogenesis and pro-tumorigenic leukocyte recruitment and can be associated with decreased survival in some cancers, such as colon cancer. On the other hand, in melanoma, high Th17-related cytokine levels in serum are associated with progression-free survival with ipilimumab [65]. It can be speculated that in tumors where IL-17 is pro-tumorigenic, and the microbiota support Th17, more autoimmune responses can be seen upon tumorigenesis.

### 3.3. Current Onco-Immunotherapies Associated with Autoimmune Responses

The breakthrough in cancer therapies in the last decade has been through cancer immunotherapy. Cancer immunotherapy approaches have shown remarkable clinical efficiency and have quickly received approval for some solid and hematological malignancies. However, this treatment modality can also induce severe side effects. Most of these side effects are immune-mediated toxicities, also known as irAEs.

The causes of irAEs are multifactorial. Since the aim in cancer immunotherapy is to increase immune activation against cancer cells, the resultant immunostimulatory milieu can activate the autoreactive lymphocytes. Furthermore, on-target toxicities wherein the antibodies or T cells target the TAAs on normal tissues can arise. Immunotherapy can also induce epitope spreading, wherein responses are raised against additional antigens to the originally targeted ones, which might include autoantigens. A by-stander effect, wherein responses to self-antigens are evoked during an immune attack directed to another target in the vicinity, can also promote treatment-associated autoimmune responses in solid cancers.

As will be discussed in the next section, the characterized autoimmune responses during tumorigenesis and tumor progression are mainly aAb-based. However, in irAEs, we observe the involvement of both autoreactive B and T lymphocytes in the form of humoral and cellular autoimmunity, a profile that is more akin to autoimmune diseases. T cell involvement has been also underscored by the association between an increase in T cell repertoire and irAE development [62,69]. However, unlike autoimmune diseases, irAEs are usually self-limiting, and they resolve upon the cessation of treatment [70]. In terms of rheumatic irAEs, aAbs such as anti-rheumatoid factor (RF) and anti-cyclic citrullinated peptide (CCP), commonly observed and used as diagnostic markers in rheumatoid diseases, are not usually observed in the sera of cancer patients [70]. Similarly, aAbs against pancreatic islet antigens are frequently present in autoimmune type I diabetes; however, they are detected less often in patients that develop diabetes as a result of ICI. Autoimmune diseases also show a strong sex bias, but in inflammatory arthritis due to ICI, gender distribution was shown to be equal [71]. Therefore, there are similarities and differences between autoimmune diseases and irAE profiles. In some cases, preexisting autoimmunity such as the anti-acetylcholine receptor antibodies seen in thymoma can also increase the risk of irAEs [69]. It is thus probable that autoimmune responses could sometimes be due to the occult autoimmunity, whereas in other instances, they are generated ‘de novo’ during cancer formation and treatment.

#### 3.3.1. ICI-Induced Autoimmune Responses

**Figure 2:**
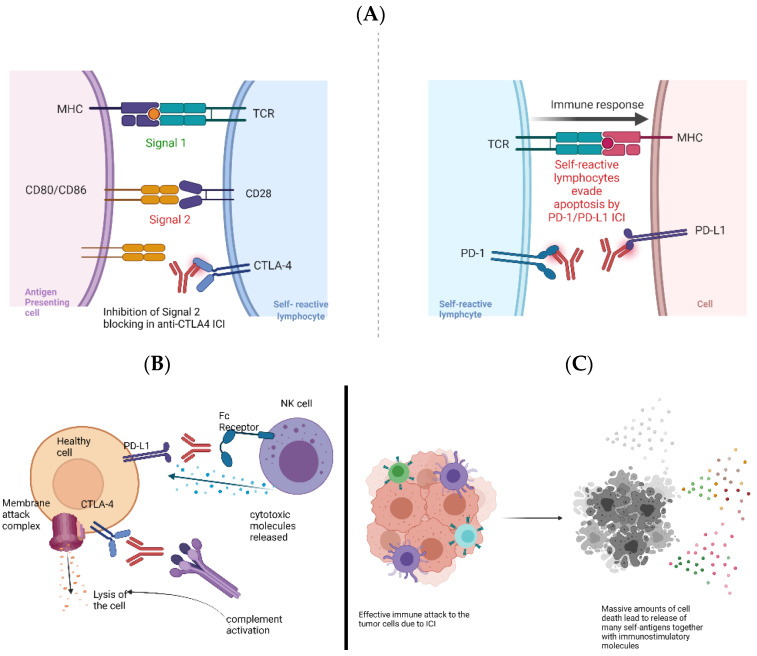
The breaking of immune tolerance in ICI therapy. (**A**) ICI can lift the inhibitory signal for the activation of self-reactive lymphocytes (left panel) or prevent the apoptosis of effector self-reactive lymphocytes (right panel). (**B**) Expression of immune checkpoint molecules on healthy cells can lead to the destruction of self-cells by antibody-mediated mechanisms such as activation of the classical complement pathway and antibody-dependent cellular cytotoxicity (ADCC). (**C**) The effective immune response against the tumor cells as a result of ICI can lead to high amounts of cell death, which results in the release of many self-antigens in a pro-inflammatory milieu. This can enable the activation of self-reactive lymphocytes, which normally have low avidity and affinity to the cognate self-antigen and a high activation threshold.

ICI has exhibited profound success and found its place in cancer treatments for various cancer types including melanoma, non-small cell lung cancer (NSCLC), and bladder cancer. Immune checkpoint molecules normally limit immune activation to prevent excessive immune-mediated damage and restore immune homeostasis. They are also important in maintaining peripheral tolerance. CTLA-4 and PD-1/PD-L1 work at different stages of T cell activation. CTLA-4 normally binds to CD80 and CD86 molecules on the antigen-presenting cells, limiting the binding of costimulatory CD28 molecules and depriving the T cells of the signal 2 required for their activation. PD-1/PD-L1, on the other hand, works more downstream and in the periphery by inducing the apoptosis of already-activated T cells. PD-1/PD-L1-based ICI can, thus, enable the function of non-exhausted effector cells and reverse the unresponsiveness of exhausted effectors [72]. Therefore, ICI works either by increasing the co-stimulation of T cells by CTLA-4-blocking monoclonal antibodies (e.g., ipilimumab) or by inhibiting the induced death of effector T cells via PD-1 (pembrolizumab and nivolumab) and/or PD-L1 (atezolizumab, avelumab) blockage. By blocking these molecules during ICI to favor anti-tumor immune responses, the breaks on the self-reactive T cells can also be lifted, leading to an immune response against normal cells (Figure 2) [73].

irAEs are noted in 60% of patients that are given ICI [74]. irAEs include, but are not limited to, dermatitis, hypophysitis, pneumonitis, pancreatitis, hepatitis, type I diabetes, colitis, and encephalitis [64]. These immune-based toxicities usually develop sometime after the initiation of treatment, which may even be after a year [75]. irAEs induced by anti-CTLA4 and anti-PD-1/PD-L1 immunotherapies can show differences in frequency. For example, colitis and diarrhea as irAE are seen more often with anti-CTLA4 therapy than with anti-PD-1/PD-L1 therapy [71]. Hypothyroidism is also seen more frequently with the former therapy; however, it has been observed most frequently in the combinatorial therapy that includes both of the checkpoint molecules [71]. These differing irAEs can be due to the different mechanisms of negative regulation normally imposed by these molecules. For example, it has been shown that anti-CTLA-4 therapy can lower the number of CTLA-4+ Tregs by ADCC, so this ICI might not only work through increasing co-stimulation, but also by directly decreasing the suppressor populations [17].

Another reason for irAE development could be the differential expression of these molecules on the body’s normal tissues, as exemplified by CTLA-4 expression in hypophyseal cells, which can lead to anti-CTLA4 related hypophysitis, as well as PD-L1 expression on pancreatic islet cells, which can induce type I diabetes upon PD-L1 targeting [69].

Combining these two effective approaches through the co-administration of CTLA-4 and PD-1/PD-L1 ICI increases the severity of irAEs and can lead to treatment discontinuation in one-third of patients [69]. This shows, once again, the importance of teasing apart the mechanistic link between the ICI and irAEs to fine tune treatments to avoid autoimmune-related toxicity and treatment discontinuation.

#### 3.3.2. Autoimmunity in Other Onco-Immunotherapy Approaches

In addition to ICI, cancer immunotherapy can include adoptive T cell transfer to increase the number of anti-tumor effector cells, cytokine administration and DC vaccines to stimulate the effector cells, and cancer vaccines, both in the form of peptides and nucleic acids, to present TAAs) to the effector cells.

Soluble components of the immune system are potent in shaping lymphocyte activation, polarization, and function. IL-2, a potent T cell activator, and interferon alpha (IFN-α) administration are used in solid cancers including melanoma, renal cell carcinoma, and colorectal cancer. These cytokine treatments trigger T cell activation and effector function non-discriminately. For example, pernicious anemia was observed upon IFN-α administration in mid-gut carcinoid tumors [73]. Vitiligo is an autoimmune hypopigmentation phenomenon due to an immune attack on melanocytes. Vitiligo development as a result of autoimmune attack to melanocytes upon IL-2 administration has also been detected and has been correlated with good treatment response [73]. IrAEs can, thus, be due to collateral damage from inflammation and immune activation upon cytokine therapy [62].

In adoptive T cell therapy, the lack of activated effector T cells in vivo is made up for by stimulating patient-derived T cells ex vivo and reinfusing them to patients [60]. To give a competitive advantage to the transferred T cells, lymphodepletion can also be carried out. However, such interventions can increase the risk of on-target autoimmune toxicities, as exemplified by ocular attacks to melanin-expressing cells and vitiligo, in melanoma adoptive immunotherapy [73]. Chimeric Antigen Receptor (CAR) T cell therapy takes adoptive immunotherapy to the next level by custom producing T-cell receptors (TCR) with a desired specificity. CAR T cell technology counteracts the lack of naturally occurring antigen-specific T cells by using gene modification to create TCRs. Currently, there are four Food and Drug Administration (FDA)-approved, CD19-specific CAR T cell therapies, but they are only for hematological malignancies. In studies using carbonic anhydrase IX-specific CAR T cells in renal cell carcinoma, grade 3–4 liver toxicities were observed, indicating the irAE problem in CAR T cell therapies for solid cancers. [76]. A very recent study used CAR technology to engineer macrophages that target HER-2 positive cancers, and these macrophages phagocytosed the tumor cells and presented antigens to T cells [77]. The upcoming clinical trials will shed light on the efficiency and the irAE risk of this approach. As exemplified by this case, the repertoire of cancer immunotherapy is increasing at a fast pace, and thus, irAEs are becoming a higher-priority problem to tackle.

### 3.4. Conventional Onco-Therapies

In addition to immunotherapy, conventional chemotherapy can also lead to autoimmune responses. Chemotherapy often targets the cell cycle to counteract uncontrolled cell growth in cancer. These approaches target all of the dividing cells nonspecifically and create massive amounts of apoptosis. The release of self-antigens during apoptosis, especially in immunogenic cell death (ICD), can create an immunogenic milieu and increase the number of peptides available for self-reactive lymphocytes, which have low avidity. In contrast to conventional apoptosis, in ICD, an immune response is generated upon cell death, which is usually associated with endoplasmic reticulum (ER) stress. In fact, one of the mechanisms of action of some chemotherapeutic agents is through ICD [78]. As an example of autoimmunity induced by chemotherapeutics, bleomycin, which induces DNA damage, can cause skin sclerosis in cancer patients [79]. Furthermore, lupus- and RA-like syndromes can also be seen with the chemotherapeutic agents and aromatase inhibitors used in estrogen receptor-positive breast cancer [70,79,80].

Likewise, in radiation therapy, the localized killing of cancer cells by ionizing radiation can stimulate systemic immune responses, as seen in the abscopal effect. The abscopal effect refers to the resolution of lesions away from the site targeted by the radiotherapy, a phenomenon that is considered to be mediated by immune activation [81]. This effect points to the immunostimulatory environment created by radiation therapy, which can aid in the evasion of immune tolerance. In terms of autoimmunity, radiation therapy has been shown to trigger new localized scleroderma in patients with SS [79].

### 3.5. Changes in Self-Antigens

As discussed in the previous subsections, aAb generation is aided by the ‘adjuvant’ effect of conventional chemotherapeutic approaches, the breaking of peripheral tolerance by cancer immunotherapy interventions, or tumor-related inflammation [82]. The targets of tumor-associated aAbs can be self-antigens that are mutated or truncated, are aberrantly expressed (in time, place, and amount), or that have different post-translational modification (PTM) patterns. Different solid cancers can also share some common aAb repertoires. This could be due to common changes in protein structure and levels across different cancers as well as the antigenic potential of certain peptides.

- **aAbs against mutated proteins:** aAbs against a commonly mutated gene product, p53, have been observed in several solid cancer types including colorectal, ovarian, lung, and breast cancer [82,83,84,85]. aAbs against some other frequently mutated proteins in cancer that have a role in cell cycle, such as c-myc and cyclin B1, are also found in some patients with solid cancers, including ovarian and lung cancer [82,86]. It is of note that these aAbs can also be observed in SLE, which is an auto-immune pathology [86]. Given the importance of apoptosis in both cancer and SLE, these aAbs might have pathophysiological importance [87].
- **aAbs against proteins with aberrant PTM:** Aberrant or modified PTMs could also induce an autoimmune response by increasing the amount of and the affinity for the presented self-peptides [88]. PTMs are a diverse set of modifications, including phosphorylation, acetylation, SUMOylation, and O-glycosylation. Glycosylation is particularly important in cell recognition, adhesion, and motility. Mucin-1 (Muc-1) is a common TAA in epithelial cancers due to its aberrant glycosylation [89]. Muc-1 aAbs have been detected with prognostic significance in lung cancer, among others [90]. In terms of other PTMs, amino acids such as aspartic acid residues can be converted to isoaspartyl residues that create neo-epitopes [91]. In oncoproteomics, state-of-the-art, high-throughput, high-content, highly reproducible, and robust screening approaches such as nucleic acid programmable protein arrays (NAPPA) and reverse phase protein arrays (RPPA), as well as mass spectroscopy techniques, are being employed to further define the aberrant PTM landscape of cancers (cancer PTMome) and its associated antibodies in cancer [88,92].
- **aAbs against cancer testis antigens and oncofetal proteins:** These proteins could also be immunogenic because of their aberrant expression in terms of location and stage of life. These antigens are normally only expressed during embryonic life and are not found in adult somatic cells. They can be re-expressed in tumor cells via processes such as DNA methylation, histone modification, or mi-RNA regulation [93]. Several examples include important TAAs such as MAGE-A1 and NY-ESO-1, which can induce immune responses in melanoma and lung cancer, respectively [94]. NY-ESO-1 expression is normally restricted to germline and embryological cells; however, it gets re-expressed in a wide range of tumors including esophageal squamous cell carcinoma, breast, lung, and prostate cancers [94]. Additionally, the presence of NY-ESO-1 aAbs was found to be a good biomarker for a better response to anti-PD-1 therapy in NSCLC [95]. Of clinical application importance, in a study where anti-NY-ESO-1 aAbs were analyzed by enzyme-linked immunosorbent assay (ELISA), aAbs were present in 7–31% of cases of esophageal cancer, lung cancer, hepatocellular carcinoma, gastric cancer, colorectal cancer, prostate cancer, and breast cancer; however, none of the healthy controls had these aAbs, making aAbs against this antigen a highly specific potential cancer biomarker [96]. A recent study that used protein microarrays to screen for the presence of 30 aAbs in nasopharyngeal cancer patients also showed that NY-ESO-1 (along with cyclin B1, survivin, and IMP3) could serve as a biomarker for the detection of this type of cancer [97]. A commercial product using NY-ESO-1, among others, is being used for early diagnosis of lung cancer in high-risk individuals. Furthermore, in protein assays using lung cancer analytes (PAULA’s test), this aAb is being assayed together with three tumor antigens for early detection of NSCLC.
- **aAbs against proteins with altered expression levels:** Examples of aAbs against the proteins that are expressed at aberrant levels are survivin as well as heat shock proteins. Survivin is a protein that inhibits apoptosis via caspase inhibition through its interacting partners [98]. This molecule also inhibits autophagy, another process that has emerged as pivotal both in cancer and autoimmune disorders. Autophagy has important physiological roles such as fine-tuning protein levels and preventing the accumulation of damaged cellular components [99]. Therefore, faults in autophagy can lead to the accumulation of damaged and/or altered proteins, which can induce autoimmune responses. Autophagy can also promote genetic instability, which provides the leeway for cancer cells to counteract treatment or immune attacks. As a protein that helps in both the evasion of apoptosis as well as autophagy, survivin is overexpressed in many solid cancers including lung and breast cancer. It is also an important molecule for the T cell receptor formation of thymocytes and differentiation into effector and memory T cells [100]. aAbs against survivin are found in both chronic hepatitis and liver carcinoma patients, pointing at a shared target molecule for both types of diseases [101].
- Heat shock proteins (HSPs) are expressed in cellular stress situations to help the cell in coping with the demand imposed by the stressor. HSPs are usually chaperones that aid in increased protein translation and/or correct folding of misfolded proteins. HSPs have anti-apoptotic properties and can help cancer cells evade apoptosis. Accordingly, they are overexpressed in a wide range of cancers, and this overexpression is a bad prognostic marker for some cancers [102,103]. Moreover, these molecules are shown to be involved in ICD. In ICD, damage-associated molecular patterns (DAMPs) are released, which ‘notify’ the immune system of the presence of harm and the need for immune responses. HSPs that are released into the extracellular environment can act as DAMPs and can induce immune responses, mainly through the activation of dendritic cells [102]. Hence, HSP 70 and 90 are being tested as cancer vaccines in breast cancer, renal carcinoma, etc. [102]. aAbs against various HSPs including HSP 70–90 have been consistently detected in cancer patients [86]. These aAbs are also observed in a wide range of autoimmune diseases including SLE and RA [86]. Anti-HSP90 aAbs in breast cancer patients are associated with a bad prognosis [103]. It was shown that an aAb against a single HSP was present in between 8%–40% of cancer patients as opposed to 1.6–25% of healthy subjects [103]. Anti-HSP aAbs are also found in some aAb panels that are being investigated for the diagnosis of various cancers including NSCLC, hepatocellular carcinoma, and prostate cancer [103].

## 4. The Significance of Autoimmune Responses in Solid Cancers

### 4.1. The Significance of Autoantibodies

aAbs can sometimes be detected months before the onset of cancer, potentially signaling an early ‘tug of war’ between the immune system and emerging cancer cells. Hence, assessing the presence of autoantibodies seems useful in the early detection of cancer. Because tumors are highly heterogeneous, the use of aAbs as biomarkers would probably be harnessed in panels that containe a combination of aAbs and/or autoantigens, rather than single aAbs, to give optimum sensitivity and specificity. Thanks to high-throughput techniques such as protein microarrays, screening, validating, and using these panels are feasible, affordable, and quick. Currently, panels of aAbs are being tested for early cancer diagnoses such as lung cancer diagnosis [83] (Table 1). Furthermore, there are already commercial blood-based tests that either exclusively screen for a panel of aAbs, such as Early CDT-lung for lung cancer diagnosis, or combine an autoantibody panel with serum protein biomarkers, such as Videssa Breast for breast cancer diagnosis.

Characterizing the targets, triggers, and effects of the aAbs could also be important for providing information on the immunogenicity profile of self-epitopes to guide the design of peptide-based cancer vaccines [7,91]. Furthermore, if these aAbs are shown to have any anti-tumor actions, they can be integrated into cancer immunotherapy techniques.

One of the fundamental questions about aAbs in a tumor is whether they serve any function for/against cancer. It could be that only tumor cells that can evade or even use these autoantibodies to their benefit are selected for in the tumor mass. aAbs against a chaperone from the HSP 70 family, GRP78, are in fact shown to bind to the aberrantly membrane-expressed form of this chaperone. GRP78 can induce an unfolded protein response and prostate cell proliferation [9]. A similar, tumor-promoting role has been suggested for aAb against an estrogen receptor, where the aAb acted as an agonist to promote breast cancer cell proliferation [109].

On the other hand, the presence of aAbs can also be an indicator of a tumor-decimating immune response. Accordingly, in the Muc-1 aAb study mentioned above, the levels of aAbs were correlated with longer survival in lung cancer patients [90]. Anti-dsDNA aAbs in colorectal cancer were also associated with better outcomes [91]. Similarly, the presence of anti-nuclear antibodies (ANAs) was associated with better survival in stage III NSCLC [91]. ANAs can bind to their exposed antigens during apoptosis and might opsonize those cells, which might facilitate anti-tumor immune responses [91]. aAbs against DNA topoisomerase I were also associated with better overall survival rates in stage I, II, and IV NSCLC [110]. Another study showed that aAbs to human DNA topoisomerase I were biomarkers for favorable prognosis in breast cancer, and this antibody induced ADCC in the in vitro studies with ER+ and triple-negative human breast cancer cell lines [111]. This study is important because it demonstrates the direct link between the aAbs and anti-tumor immune responses. Another study has also shown that the presence of an aAb to topoisomerase I, as detected by ELISA in the absence of survivin-expressing cancer cells (in the peripheral blood), is associated with longer survival in endometrial cancer as compared to patients without aAbs but with survivin-positive cancer cells in the peripheral blood [112].

The presence of aAbs can also be associated with the mutational load in tumoral cells. It was shown that the higher the mutational load of the tumor (‘hot tumors’), the better the response to ICI [113]. A plausible explanation for this observation is the formation of neoantigens in hot tumors, which can then be recognized by effector T cells without the constraints of central tolerance faced by self-antigens [114]. The presence of aAbs can thus be an indirect indicator of higher mutational load, clarifying the link between the presence of some aAbs and treatment response.

In addition to the prognostic and tumor-centered importance of aAbs in cancer, another obvious question about these aAbs is whether they cause autoimmune damage to the other structures of the body via various mechanisms. This damage can arise when the immune system tries to clear the immune complexes through the Fc receptors present on the phagocytic cells or when the complement cascade is activated via these immunoglobulins, among other mechanisms. Clustering of some autoimmune diseases with cancers can be associated with destructive autoimmune responses. Systemic sclerosis (scleroderma) is an autoimmune disease that presents with widespread vasculopathy and fibrotic changes. Interestingly, the individuals that have aAbs against the RNA polymerase III subunit can have temporal clustering of scleroderma and cancer [115]. The question arises whether these aAbs lead to a scleroderma-inducing autoimmune attack. Interestıngly, this phenomenon is not detected in the presence of topoisomerase 1 or centromere protein B aAbs [115]. The same study showed that there were somatic mutations or loss of heterozygosity in 6 out of 8 patients in the gene coding for RNA polymerase III (*POL3RA*), pointing at the mutated protein as the cause of aAbs. A similar occurrence was seen in dermatomyositis, where the presence of anti-NXP2 and anti-transcription intermediary factor-1 gamma aAb correlated with cancer development right before or after dermatomyositis onset [79,116]. Furthermore, the progress of the dermatomyositis paralleled the progress of cancer, with co-relapses or co-resolutions [79]. It can be speculated that these aAbs could be the remnants of an early, failed anti-tumor immune response that led to an autoimmune pathology [7].

Another autoimmune attack in cancer is seen in paraneoplastic syndrome. This syndrome is a rare occurrence (approximately 1 in 10,000 patients with cancer) and shows symptoms not directly attributable to the tumor presence [11]. Paraneoplastic syndrome, as its name suggests, can occur before or right after the clinically overt cancer. Paraneoplastic syndromes are thought to be mediated by the tumoral secretion of functional molecules such as hormones and/or immune attacks due to the cross-reactivity of the anti-tumoral immune responses [11]. The latter is especially important in neurologic paraneoplastic syndromes, with Lambert-Eaton myasthenic syndrome (LEMS) occurring in 1% of patients with small-cell lung cancer [117]. Classic neurological paraneoplastic syndromes also include encephalitis and cerebellar ataxia [118]. The targets of aAbs in neurological paraneoplastic syndromes usually include onco-neuronal antigens such as Hu antigen or anti-amphiphysin [117]. The presence of anti-Hu aAbs and paraneoplastic neuropathies and encephalopathies are associated with better response to therapy, pointing to a potential cross-reactivity between neuronal antigens and TAAs [64].

**Figure 3:**
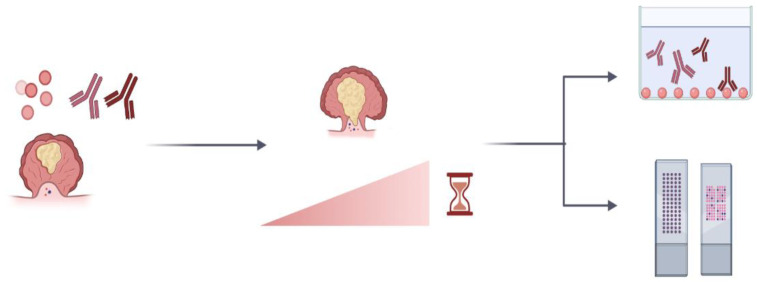
Advantages of autoantibodies as tumor biomarkers. Autoantibodies can be present at very early stages of disease to help in life-saving early diagnosis of cancer. They are stable molecules which facilitates their detection. Autoantibodies can easily be assayed by conventional and widespread techniques. The parallel detection of multiple autoantibodies is also possible with protein microarrays to increase specificity and sensitivity.

Overall, aAbs have many advantages as tools for the early diagnosis of various solid cancers (Figure 3). Their biological and prognostic capabilities are probably dependent on their specificity, their immunological properties, and the immune milieu of the body and cancer.

### 4.2. The Significance of irAEs

As mentioned before, irAEs can limit the clinical utility of ICIs, leading to treatment discontinuation due to severe toxicities. Therefore, the biggest challenge in cancer immunotherapy is to prevent trading one evil for another in the form of autoimmunity. Thus, while eliminating cancer using immune components, destructive and potentially lethal autoimmunity should ideally be avoided. Steroid administration to counteract irAE does not seem to reduce the treatment efficiency of ICI, giving hope that different cell populations are involved in these two events, and irAEs are not obligate side effects of ICI.

Autoimmunity induced by ICI can also be associated with a good treatment response similar to some autoantibodies mentioned before, as it is often observed in melanoma. After ipilimumab treatment, Melan-A-specific cytotoxic T cells increase in peripheral blood, in tumor tissue, and in autoimmunity-induced skin rashes [73]. Vitiligo has been shown that it is associated with progression-free survival and overall survival in patients with advanced melanoma receiving immunotherapy, pointing to lymphocytes that are cross-reacting to melanoma and normal melanocytes [71]. Cutaneous irAEs (vitiligo, pruritus, nonspecific macular rash, etc.) are also observed frequently. When an analysis was carried out through grouping all cutaneous irAEs, they were shown to be associated with better therapeutic response and overall survival upon immunotherapy [74]. Furthermore, the presence of irAEs after nivolumab treatment was positively associated with overall survival in 143 melanoma patients [64]. A similar trend has also been observed in NSCLC, where the presence of irAEs after nivolumab and pembrolizumab regimens correlated with a good treatment response and progression-free survival [62,64]. These data might suggest that in some cases, the presence of irAEs is an indicator of the inflammatory response and heightened immune reactivity against tumors.

## 5. Conclusions and Perspectives

Autoimmune responses observed in tumor settings and solid cancer immunotherapy are complex. They can arise due to genetic changes, microbiota-related factors, TAAs, epitope spreading, the imbalance of antitumor immunity, and immune toxicity. It should also be noted that the infection history of the host and, in the specific case of virus-related cancers such as HPV-related cervical cancer, viral factors and anti-viral immunity can also potentially play a role in these responses.

Autoimmune responses in the form of aAbs can serve as stable, easy to assay, and specific biomarkers for cancer diagnosis that only require a small volume of patient samples. Advances in high-throughput proteomics in the form of protein microarrays, SEREX, and NAPPA arrays enable the screening of large cohorts of patients in parallel in a relatively short time. This could also lead to the use of aAb panels to discriminate among different tumor stages and grades, for assessing treatment responses, and for shaping treatment choices. Large prospective studies that are standardized in terms of design, the methods of detection, cut-points, and case characteristics are also important to be able to compare results across studies.

In addition to serum, the analysis of aAbs in body fluids other than peripheral blood, such as cerebrospinal fluid for glioma, can also expand the clinical utility of aAbs in clinical practice [119]. Furthermore, investigating classes of antibodies other than IgG such as IgA, especially in cancers such as colon cancer where mucosal immunity is altered, can shed more light on the autoimmune phenomenon in cancer settings.

It is of note that the relationship between autoimmunity and cancer is bidirectional, and certain autoimmune diseases such as SLE, RA, and SS increase the risk of cancers. This effect is probably mediated by chronic inflammation and the treatments involved in these diseases. The cancer to autoimmune response link, however, is mediated mainly by the autoantigens in the tumor cells, epitope spreading, and the treatment-induced, inflammatory tumor environment. However, clearly elucidating the root causes of this link would also shed light on the general concepts of immune tolerance as well as basic immunology in autoimmune diseases.

The need to understand autoimmune responses in cancer is more pressing than ever with the more widespread use of cancer immunotherapy approaches in the clinic, as irAEs are the major roadblock to their widespread use. A very recent study in a xenoplant, triple-negative breast cancer mouse model showed that targeting CD6 via an antibody decreased tumor growth and led to the activation of CD8+ and NK cells, whereas the same antibody dampened autoimmunity in mice [120]. In this way, selective activation of only anti-tumor effectors seemed possible. Similar efforts in the field would help in the fine-tuning of cancer immunotherapy approaches to evade or minimize irAEs while inducing an effective anti-tumor immune response to maximize the clinical benefits of immunotherapy.

## Figures and Tables

**Table 1:** Autoantibody panels being tested for diagnostic use in various solid cancers. (ELISA: enzyme-linked immunosorbent assay).

Type of Cancer	Comparison Group	Antibody Panel	Method Used	Sensitivity (%)	Specificity (%)	Reference
Breast cancer	Breast cancer patients vs. healthy donors	p53/PRDX6/c-Myc/Hsp70/Nm23	ELISA	34	100	[85]
Lung cancer	Patients with recent diagnosis of lung cancer vs. healthy controls	p53, NY-ESO-1, CAGE, GBU4–5, MAGE A4, SOX2, and Hu-D	ELISA (*Early* CDT-Lung)	41	93	[104]
Lung cancer	Lung cancer patients vs. healthy controls and lung benign disease group	p53,PGP9.5, SOX2, GAGE7, GBU4–5, MAGE A1, and CAGE	ELISA	25.4	91.7	[83]
Ovarian cancer	Early (stage I-II) stage ovarian cancer patients vs. healthy controls	p53, GNAS, and NPM1	ELISA	57	86	[105]
	Late-stage (stage III–IV) ovarian cancer patients vs. healthy controls			49	86	
Ovarian cancer	Ovarian cancer patients vs. healthy controls	p53, PTPRA, and PTGFR	Luminex bead assay	23.3	98.3	[106]
Colorectal cancer	Colorectal cancer patients vs. healthy individuals and breast and lung cancer patients	p53, GTF2B, MAPKAPK3, PIM1, PKN1, SRC, STK4, and SULF1	Luminex bead assay and electrochemical immunosensing by HaloTag fusion	76.0	98.6	[107]
Melanoma	Early stage melanoma patients vs. healthy controls	p53, ZBTB7B, PRKCH, PCTK1, PQBP1, UBE2V1, IRF4, MAPK8_tv2, MSN, and TPM1	Protein microarray	79	84	[108]
Nasopharyngeal carcinoma	Nasopharyngeal cancer patients vs. healthy individuals	cyclin B1, NY-ESO-1, survivin and IMP3	Protein microarray	54	86	[97]

## Data Availability

Not applicable.
